# A Convex Constraint Variational Method for Restoring Blurred Images in the Presence of Alpha-Stable Noises

**DOI:** 10.3390/s18041175

**Published:** 2018-04-12

**Authors:** Zhenzhen Yang, Zhen Yang, Guan Gui

**Affiliations:** 1 National Engineering Research Center of Communication and Network Technology, Nanjing University of Posts and Telecommunications, Nanjing 210003, China; yangzz@njupt.edu.cn (Z.Y.); yangz@njupt.edu.cn (Z.Y.); 2 Key Lab of Broadband Wireless Communication and Sensor Network Technology, Nanjing University of Posts and Telecommunications, Ministry of Education, Nanjing 210003, China

**Keywords:** image deblurring, variational method, alpha-stable noise, primal-dual algorithm, total variational

## Abstract

Blurred image restoration poses a great challenge under the non-Gaussian noise environments in various communication systems. In order to restore images from blur and alpha-stable noise while also preserving their edges, this paper proposes a variational method to restore the blurred images with alpha-stable noises based on the property of the meridian distribution and the total variation (TV). Since the variational model is non-convex, it cannot guarantee a global optimal solution. To overcome this drawback, we also incorporate an additional penalty term into the deblurring and denoising model and propose a strictly convex variational method. Due to the convexity of our model, the primal-dual algorithm is adopted to solve this convex variational problem. Our simulation results validate the proposed method.

## 1. Introduction

Noise interferences often occur in many systems such as wireless communications [1] and social networks [2,3]. Hence, images are inevitably corrupted by both blur and noise during the acquisition and transmission. Hence, the restoration of clean images from blurred and noisy observations is a fundamental task in the image processing community. A wide range of approaches has been proposed to remove additive Gaussian noise [4,5,6]. However, many other noises, such as impulse noise [7,8,9,10,11,12], multiplicative noise [13,14], Poisson noise [15,16,17], Cauchy noise [18,19], and Rician noise [20], commonly appear in the real world and thus are studied by many researchers. Another impulsive noise is often caused by alpha-stable noise, which normally appears in many applications, such as wireless communication systems, synthetic aperture radar (SAR) images, biomedical images, and medical ultrasound images [21,22].

Mathematically, the image restoration problem can be expressed as

(1) f=Ku+η

where $u\in R^{mn}$ is obtained from a two-dimensional pixel-array with dimension m×n and defined on a connected bounded domain $\Omega \subset R^{2}$ with compact Lipschitz boundary, $K\in R^{mn\times mn}$ denotes a known linear and continuous blurring operator, η is the noise obeys certain distribution (for example alpha-stable noise is the noise which obeying alpha-stable distribution), and $f\in R^{mn}$ is the blurred image with the additive noise. In particular, when *f* is corrupted only by noise, it is then given by f=u+η.

It is well known that restoring *u* from *f* is normally an ill-conditioned problem. Variational methods are proposed to handle this ill-posed inverse imaging problems. These methods are usually summarized as convex and non-convex methods, respectively. The total variation (TV) regularization method [23] plays a significant role in convex variational-based image processing, since it can preserve sharp edges in images due to the piecewise smooth property of the TV norm.

The ROF (Rudin Osher and Fatemi) denoising model is one of the most famous total variational models for restoring images with additive Guassian noise, which was proposed by Rudin et al. [6], as given by

(2) $$\underset{u\in BV\left(\Omega \right)}{\inf}\int_{\Omega }\left|Du\right|+\frac{\lambda }{2}\int_{\Omega }{\left(u-f\right)}^{2}dx\;$$

where $\int_{\Omega }\left|Du\right|$ is the TV regularization term, BV is the space of the functions of bounded variation, $\int_{\Omega }{\left(u-f\right)}^{2}dx$ is the data fidelity term, and λ>0 is the regularization parameter, which represents the trade-off between the data fidelity term and the TV regularization term. It is possible to modify the ROF denoising model to incorporate a linear blurring operator *K* [6]. The ROF deblurring and denoising model is then given as follows:

(3) $$\underset{u\in BV\left(\Omega \right)}{\inf}\int_{\Omega }\left|Du\right|+\frac{\lambda }{2}\int_{\Omega }{\left(Ku-f\right)}^{2}dx.$$

Although the ROF deblurring and denoising model is a very useful deblurring and denoising approach with additive Gaussian noise, it does not achieve good performance in the scenario of non-Guassian environments. As a result, many kinds of variational models based on TV have been proposed for restoring clean images from blurred and non-Guassian noise distribution, such as that of impulse noise [7,8,9,10,11,12], multiplicative noise [13,14], Poisson noise [15], Cauchy noise [18,19], and Rician noise [20]. Based on different noise distributions, and data fidelity terms, one can obtain appropriate variational models for image denoising and deblurring in the presence of different noises. For example, $\int_{\Omega }\left|Ku-f\right|dx$ is the data fidelity term of TVL1 deblurring and denoising model with impulse noise [11], and $\int_{\Omega }\log\left(\gamma^{2}+{\left(Ku-f\right)}^{2}\right)dx$ is the data fidelity term of Cauchy deblurring and denoising model with Cauchy noise [18].

Recently, some methods have been considered to mitigate alpha-stable noise. For example, Zozor et al. [24] employed a parametric approach for suboptimal signal detection. They dealt with the detection of a known signal embedded in alpha-stable noise and discussed the robustness of the detector against the signal amplitude and the stability index. Sadreazami et al. [25] modeled the contourlet coefficients of noise-free images with the alpha-stable distribution. They have also presented a new approach for despeckling SAR images and a multiplicative watermark detection in the contourlet domain using the alpha-stable distribution [26,27]. Yang et al. [28] proposed a total variational method to restore images that are degraded by alpha-stable noise based on the property of meridian distributed.

Until now, to the best of our knowledge, there is no paper reporting on a variational method for blurred image restoration in the presence of alpha-stable noise. In order to restore images from blur and alpha-stable noise while also preserving their edges, this paper proposes a novel variational method based on the statistical property of meridian distribution and the TV, and our numerical experiments demonstrate that it performs better than many standard deblurring and denoising method in impulsive noisy environments (with small α values, i.e., $\alpha \in \left(0,1.5\right)$), while providing comparable or better performance in less demanding, light-tailed environments (with high α values, i.e., $\alpha \in \left(1.5,2\right)$).

The main contributions of this paper are summarized as follows. (i) Based on the statistical properties of meridian distribution and the TV, we propose a new variational method for restoring blurred images with alpha-stable noise and then analyze the existence of the solution for the variational model. (ii) By adding a penalty term, we propose a strictly convex variational method and prove the existence and uniqueness of the solution for the convex variational model. (iii) The primal-dual algorithm is employed to solve the novel convex variational problem, with its convergence being analyzed. (iv) We compare our proposed method to state-of-the-art methods such as the TVL1 model [11], the Cauchy model [18], and the meridian filter [29] and show the effectiveness of our proposed method.

The rest of this paper is organized as follows. In Section 2, we describe the alpha-stable and the meridian distributions. In Section 3, we propose a variational method for simultaneous deblurring and denoising, and study the existence of the solution for the proposed model. We also propose a convex variational method to restore blurred images with alpha-stable noise, and analyze the existence and uniqueness of the solution for the convex variational model. The primal-dual algorithm for solving the proposed convex restoration problems is given in Section 4. Section 5 presents extensive numerical results to evaluate the performance of the proposed method in comparison with well-known methods. Finally, concluding remarks are provided in Section 6.

## 2. A Brief Review of the Alpha-Stable and Meridian Distributions

The alpha-stable noise which obeys alpha-stable distribution is often found in radar- and sonar-related applications. The heaviness of the alpha-stable distribution tails is controlled by the parameter α∈(0,2), namely, the tails grow thicker as α values becomes smaller. Hence, alpha-stable noise can be seen as a type of impulsive noise with small α values ($\alpha \in \left(0,1.5\right)$) [21].

The alpha-stable distributions are closed under additions, i.e., the sum of two alpha-stable random variables is still an alpha-stable random variable. Moreover, the alpha-stable random variables obey the generalized central limit theorem [21]. However, this class of alpha-stable distribution random variables has no closed-form expressions for densities and distribution functions (except for Gaussian distribution, Cauchy distribution, and Levy distribution). The distribution with α=2 corresponds to the well-known Gaussian distribution, and the one with α=1 corresponds to the Cauchy distribution.

**Figure 1:**
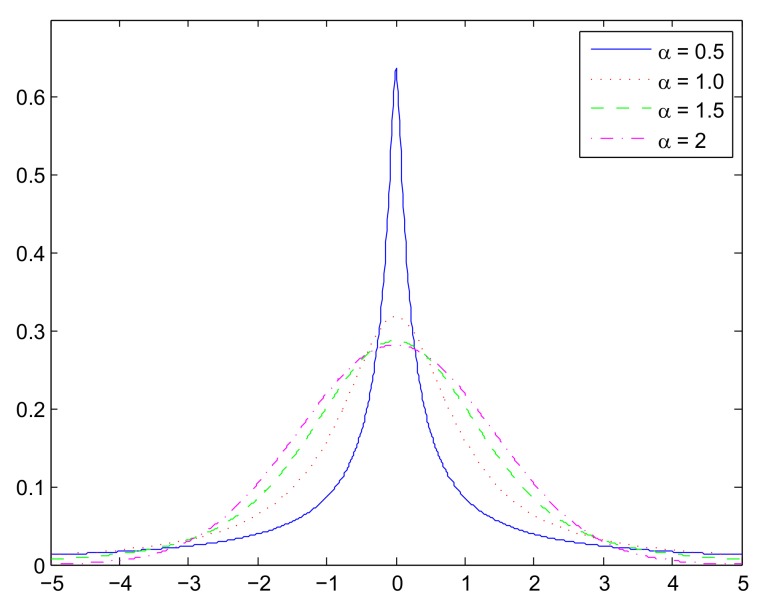
Probability density functions (PDFs) of alpha-stable distributions $S\left(\alpha ,0,1,0\right)$ with different values of α.

Figure 1 shows the probability density functions (PDFs) of alpha-stable distributions $S\left(\alpha ,0,1,0\right)$ with different values of α. We can see that the distributions of this class are all bell-shaped, with increasing density on the left and decreasing on the right. In addition, the tail of the bells becomes heavier as the value of α decreases.

The meridian distribution is a member of the generalized Cauchy distributions (GCD) family [30], and it combines the advantages of the GCD and alpha-stable distributions. Moreover, an estimator derived from the meridian distribution is robust to the impulsive noise [30]. The probability density function (PDF) of the meridian distribution is given by

(4) $$p_{M}\left(x\right)=\frac{\gamma }{2}\frac{1}{{\left(\gamma +\left|x-\theta \right|\right)}^{2}}$$

where γ>0 is the scale parameter, and θ is the localization parameter. Without loss of generality, we consider θ=0 in our paper. A careful inspection of the meridian distribution shows that its PDF tail decays slower than the Cauchy case, resulting in a heavier-tailed PDF, that is, the meridian PDF exhibits tails heavier than that of the Cauchy PDF [29]. Moreover, by examining the well-established statistical relation between the Laplacian and meridian distributions, we can find that the ratio of two independent Laplacian distributed random variables is a meridian distribution [29].

The influence function of the meridian distribution is given by

(5) $$\psi \left(x\right)=\frac{\mathrm{sgn}\left(x\right)}{\gamma +\left|x\right|}$$

where sgn(·) is the sign function. The influence function determines the effect of contamination. The rejection point of the meridian is smaller than that of the Cauchy distribution as it has a higher influence function decay rate. This indicates that a signed detection algorithm in the presence of the impulsive noise with the meridian distribution is more robust than that in the Cauchy distributed noise [29].

## 3. The Proposed Variational Model

In this section, we propose a new variational model for restoring blurred images under the alpha-stable noise environments.

Motivated by existing work [6,13,18,29], we propose a variational model by applying the Bayes rule and the maximum a posteriori (MAP) estimator to restore the blurred images with alpha-stable noise based on the property of the meridian distribution and the TV.

First, we focus only on the denoising scenario. Given a known image *f*, as in [6,13], by using the Bayes rule as well as the MAP estimation, we have

(6) $$\begin{array}{cc} \hat{u}\left(f\right) & =\arg\max_{u}P\left(u|f\right)=\arg\max_{u}\frac{P\left(f|u\right)P\left(u\right)}{P\left(f\right)}\\ & =\arg\min_{u}-\log\left(P\left(f|u\right)\right)-\log\left(P\left(u\right)\right)+\log\left(P\left(f\right)\right)\\ & =\arg\min_{u}-\log\left(P\left(f|u\right)\right)-\log\left(P\left(u\right)\right) \end{array}$$

In obtaining Equation (6), we have omitted $\log\left(P\left(f\right)\right)$ since it is a constant respect to *u*.

As the image is corrupted by alpha-stable noise, for each pixel x∈Ω, we have

(7) $$P\left(f\left(x\right)|u\left(x\right)\right)=\frac{\gamma }{2}\frac{1}{{\left(\gamma +\left|u\left(x\right)-f\left(x\right)\right|\right)}^{2}}$$

where γ>0 stands for the scale parameter. Therefore,

(8) $$\begin{array}{cc} -\log\left(P\left(f|u\right)\right) & =-\int_{\Omega }\log\left(P\left(f\left(x\right)|u\left(x\right)\right)\right)dx\\ & =\int_{\Omega }\left(2\log\left(\gamma +\left|u\left(x\right)-f\left(x\right)\right|\right)+\log2-\log\gamma \right)dx \end{array}.$$

Inspired by the idea of Aubert et al. [13], *u* is assumed to follow a Gibbs prior distribution. Therefore, we can obtain the TV regularization of *u* as follows:

(9) $$-\log\left(P\left(u\right)\right)=\int_{\Omega }\log\left(P\left(u\left(x\right)\right)\right)dx=\int_{\Omega }\left(\beta \left|Du\left(x\right)\right|+\log R\right)dx\;$$

where β>0 is a parameter, and *R* is the normalization factor. Hence, solving Equation (6) is equivalent to find the minimization of the following logarithmic probability. That is,

(10) $$\begin{array}{cc} -\log\left(P\left(f|u\right)\right)-\log\left(P\left(u\right)\right) & =-\int_{\Omega }\log\left(P\left(f\left(x\right)|u\left(x\right)\right)\right)dx-\int_{\Omega }\log\left(P\left(u\left(x\right)\right)\right)dx\\ & =\int_{\Omega }2\log\left(1+\frac{\left|u\left(x\right)-f\left(x\right)\right|}{\gamma }\right)dx\\ & \;\;\;\;+\int_{\Omega }\left(\beta \left|Du\left(x\right)\right|+\log2+\log\gamma +\log R\right)dx \end{array}.$$

Here, please note that the log2+logγ+logR is omitted since the three terms are all constants with respect to *u*.

Therefore, our pure denoising with alpha-stable noise is given by

(11) $$\underset{u\in BV\left(\Omega \right)}{\inf}E\left(u\right):=\int_{\Omega }\left|Du\right|+\lambda \int_{\Omega }\log\left(1+\frac{\left|u-f\right|}{\gamma }\right)dx$$

where $\lambda =\frac{2}{\beta }>0$ is a regularization parameter. As one can see, we keep the same regularization term as in the ROF denoising model (Equation (2)) since the TV regularization term is useful for preserving edges, but we adapt the data fidelity term to the alpha-stable noise, introducing one that is suitable for such noise. We emphasize that the proposed model can be extended to other modern regularization terms such as framelets, sharelets, rank surrogates, dictionary learning, or the tight-frame approach. These regularization terms are effective for the restoration of blurred and noisy images.

Thus, we start to prove the existence of the solution for Equation (11).

**Theorem** **1.** Let $f\in L^{\infty }\left(\Omega \right)$ with $\underset{\Omega }{\inf}f>0$, then Equation (11) has a solution $u^{*}\in BV\left(\Omega \right)$ satisfying:

$$0<\underset{\Omega }{\inf}f\leq u^{*}\leq \underset{\Omega }{\sup}f.$$

**Proof.**  Set $a=\underset{\Omega }{\inf}f$, $b=\underset{\Omega }{\sup}f$, and let $E_{0}\left(u\right):=\lambda \int_{\Omega }\log\left(1+\frac{\left|u-f\right|}{\gamma }\right)dx$. Noting that $E\left(u\right):=\int_{\Omega }\left|Du\right|+\lambda \int_{\Omega }\log\left(1+\frac{\left|u-f\right|}{\gamma }\right)dx$, we have $E\left(u\right)\geq E_{0}\left(u\right)\geq 0$. This leads to $E\left(u\right)$ being lower-bounded, and we can find a minimal sequence $\left\{u_{n}\right\}\subset BV\left(\Omega \right)$.

In addition, for any fixed x∈Ω, let $h\left(t\right):=\log\left(1+\frac{\left|t-f\left(x\right)\right|}{\gamma }\right)$. Therefore, if $t>f\left(x\right)$, we have $h^{\prime }\left(t\right)=\frac{1}{\gamma +t-f\left(x\right)}>0$, else if $t<f\left(x\right)$, we get $h^{\prime }\left(t\right)=-\frac{1}{\gamma +f\left(x\right)-t}<0$. From the above two inequalities, we know that the function h(t) is decreasing if $t\in \left[0,f\left(x\right)\right]$ and increasing if $t\in \left[f\left(x\right),+\infty \right)$. This implies that $h\left(\min\left(t,M\right)\right)\leq h\left(t\right)$ if $M\geq f\left(x\right)$. Hence, $E_{0}\left(\underset{\Omega }{\inf}\left(u,b\right)\right)\leq E_{0}\left(u\right)$ if M=b. Furthermore, it is known that $\int_{\Omega }\left|D\underset{\Omega }{\inf}\left(u,b\right)\right|\leq \int_{\Omega }\left|Du\right|$ (see Lemma 1 in [31]). Therefore, we can conclude that $E\left(\underset{\Omega }{\inf}\left(u,b\right)\right)\leq E\left(u\right)$. Similarly, $E\left(\underset{\Omega }{\sup}\left(u,a\right)\right)\leq E\left(u\right)$ with $a=\underset{\Omega }{\inf}f$. Hence, we can assume that $0<a\leq u_{n}\leq b$, which implies that $u_{n}$ is bounded in $L^{1}\left(\Omega \right)$.

According to the definition of $\left\{u_{n}\right\}$, $E\left(u_{n}\right)$ is bounded. In addition, it is proved that $u_{n}$ is bounded in $BV\left(\Omega \right)$ since $\int_{\Omega }\left|Du_{n}\right|$ is bounded [31]. Hence, there is a subsequence that converges strongly in $L^{1}\left(\Omega \right)$ and weakly in $BV\left(\Omega \right)$ to some $u^{*}\in BV\left(\Omega \right)$. Furthermore, given $0<a\leq u^{*}\leq b$, the lower semicontinuity of the TV, and the Fatou’s Lemma, the solution to Equation (11) is obtained as $u^{*}$. ☐

We then extend Equation (11) to the simultaneous deblurring and denoising scenarios. The restoration is conducted by solving the following optimization model:

(12) $$\underset{u\in BV\left(\Omega \right)}{\inf}\int_{\Omega }\left|Du\right|+\lambda \int_{\Omega }\log\left(1+\frac{\left|Ku-f\right|}{\gamma }\right)dx.$$

It is worth mentioning that Equation (12) is also a non-convex problem, as in the scenario of the pure denoising Equation (11). Since Equations (11) and (12) are both nonconvex, they cannot guarantee a global optimal solution. To overcome this drawback, we incorporate an additional penalty term into Equations (11) and (12) to obtain novel convex variational models in the following section. This penalty term is based on the median-filtered result of the noise image.

In the following section, we propose a convex variational model for deblurring and denoising images, which is corrupted by both blur and alpha-stable noise.

We first also focus on a convex variational model for denoising only. By introducing a penalty term into Equation (11), we obtain a convex variational model as follows:

(13) $$\underset{u\in BV\left(\Omega \right)}{\inf}\int_{\Omega }\left|Du\right|+\lambda \left(\int_{\Omega }\log\left(1+\frac{\left|u-f\right|}{\gamma }\right)dx+\frac{\mu }{2}{∥u-g∥}_{2}^{2}\right)$$

where $g=\mathrm{medfilt}2\left(f\right)$ (*g* is the median filter function of *f* ) [18], λ>0 and μ>0 are the regularization parameters, respectively.

As a result, three theorems are provided to confirm that the above model is strictly convex under certain conditions, and there is a unique solution to Equation (13).

**Lemma** **1.** If $\mu \gamma^{2}\geq 1$, the objective function in Equation (13) is strictly convex.

**Proof.**  For each fixed x∈Ω, let the real function *h* on $R^{+}\cup \left\{0\right\}$ be defined as

$$h\left(t\right):=\log\left(1+\frac{\left|t-f\left(x\right)\right|}{\gamma }\right)+\frac{\mu }{2}{\left(t-g\left(x\right)\right)}^{2}.$$

We can easily compute the first and second order derivatives of *h*, as given by

$$h^{\prime }\left(t\right)=\frac{\mathrm{sgn}\left(t-f\left(x\right)\right)}{\gamma +\left|t-f\left(x\right)\right|}+\mu \left(t-g\left(x\right)\right)$$

$$h^{\prime \prime }\left(t\right)=-\frac{\left|\mathrm{sgn}\left(t-f\left(x\right)\right)\right|}{{\left(\gamma +\left|t-f\left(x\right)\right|\right)}^{2}}+\mu .$$

Since $\mu \gamma^{2}\geq 1$, we have $\gamma \geq \frac{1}{\sqrt{\mu }}$; thus, $\gamma +\left|t-f\left(x\right)\right|\geq \frac{1}{\sqrt{\mu }}$, or $\mu {\left(\gamma +\left|t-f\left(x\right)\right|\right)}^{2}\geq 1$, that is $h^{\prime \prime }\left(t\right)\geq 0$, i.e., *h* is convex. Furthermore, the function *h* has only one minimizer, so *h* is strictly convex when $\mu \gamma^{2}\geq 1$. Since the total variation regularization is convex, we can also conclude that the objective function in Equation (13) is strictly convex for $\mu \gamma^{2}\geq 1$. ☐

Based on Lemma 1, we can now prove the existence and uniqueness of the solution to Equation (13).

Lastly, we also extend our convex variational model for the following simultaneous deblurring and denoising case:

(14) $$\underset{u\in BV\left(\Omega \right)}{\inf}\int_{\Omega }\left|Du\right|+\lambda \left(\int_{\Omega }\log\left(1+\frac{\left|Ku-f\right|}{\gamma }\right)dx+\frac{\mu }{2}{∥Ku-g∥}_{2}^{2}\right).$$

Since the blurring operator *K* is linear and nonnegative, we can conclude that the model in Equation (14) is convex when $\mu \gamma^{2}\geq 1$. In the following theorem, we state the existence and uniqueness of its solution.

**Theorem** **2.** Let $f\in L^{\infty }\left(\Omega \right)$ with $\underset{\Omega }{\inf}f>0$, $g\in L^{2}\left(\Omega \right)$, and a nonnegative linear operator $K\in L\left(L^{1}\left(\Omega \right),L^{2}\left(\Omega \right)\right)$. Assume that K does not annihilate constant functions, i.e., KI≠0. Therefore, Equation (14) has a solution. Further, if $\mu \gamma^{2}\geq 1$ and K is injective, the solution is unique.

**Proof.**  Let $\left\{u_{n}\right\}\in BV\left(\Omega \right)$ be a minimizing sequence for Equation (14). Since the objective function in (14) is bounded, we know that $\left\{\int_{\Omega }\left|Du_{n}\right|\right\}$ is bounded [13,18]. As in the proof of Theorem 2 of [18], we can verify that ${∥u_{n}-m_{\Omega }\left(u_{n}\right)∥}_{2}$ and ${∥u_{n}-m_{\Omega }\left(u_{n}\right)∥}_{1}$ are bounded for each *n* (where $m_{\Omega }\left(u_{n}\right)=\frac{1}{\left|\Omega \right|}\int_{\Omega }u_{n}dx$, $\left|\Omega \right|$ denotes the measure of Ω). Due to the continuity of the operator $K\in L\left(L^{1}\left(\Omega \right),L^{2}\left(\Omega \right)\right)$, we know that the sequence $\left\{K\left(u_{n}-m_{\Omega }\left(u_{n}\right)\right)\right\}$ is bounded in $L^{2}\left(\Omega \right)$ and in $L^{1}\left(\Omega \right)$.

Moreover, for each *n*, the objective function in Equation (14) is bounded, hence ${\left(Ku_{n}-g\right)}^{2}$ is bounded in $L^{1}\left(\Omega \right)$. Thus, ${∥Ku_{n}-g∥}_{1}$ is bounded as well, and hence ${∥Ku_{n}∥}_{1}$ is bounded. One can easily find that $\left|m_{\Omega }\left(u_{n}\right)\right|{∥K1∥}_{1}$ is bounded from Equation (15).

(15) $$\begin{array}{cc} \left|m_{\Omega }\left(u_{n}\right)\right|{∥K1∥}_{1} & ={∥K\left(u_{n}-m_{\Omega }\left(u_{n}\right)\right)-Ku_{n}∥}_{1}\\ & \leq {∥K\left(u_{n}-m_{\Omega }\left(u_{n}\right)\right)∥}_{1}+{∥Ku_{n}∥}_{1} \end{array}.$$

Since K1≠0, $m_{\Omega }\left(u_{n}\right)$ is uniformly bounded. Moreover, $\left\{u_{n}-m_{\Omega }\left(u_{n}\right)\right\}$ is bounded, so $\left\{u_{n}\right\}$ is bounded in $L^{2}\left(\Omega \right)$ and in $L^{1}\left(\Omega \right)$. Since $BV\left(\Omega \right)$ is closed and convex, $\left\{u_{n}\right\}$ is also bounded in $BV\left(\Omega \right)$.

As a consequence, there is a possible subsequence $\left\{u_{n_{k}}\right\}$, which converges in $L^{1}\left(\Omega \right)$ to some $u^{*}\in BV\left(\Omega \right)$, and $\left\{Du_{n_{k}}\right\}$ converges slightly as a measure to $Du^{*}$. Since the linear operator *K* is continuous, $\left\{Ku_{n_{k}}\right\}$ converges to $Ku^{*}$ in $L^{2}\left(\Omega \right)$. Thus, $u^{*}$ is a solution of Equation (14) according to the lower semicontinuity of TV and Fatou’s lemma.

Based on Lemma 1, when $\mu \gamma^{2}\geq 1$, Equation (14) is strictly convex. Furthermore, *K* is injective, so its solution is unique. ☐

## 4. Primal-Dual Algorithm

In this section, we employ the primal-dual algorithm [32,33] to solve the minimization problem in (14) since it is easy to implement and its convergence is guaranteed [32]. Due to the convexity of Equation (14), there are many algorithms that can be employed to solve the proposed image deblurring and denoising model such as the alternating direction method of multipliers (ADMM) [5,34,35] and the split-Bregman algorithm [36].

We address the general deblurring and denoising case, since the pure denoising case can be considered special when *K* is an invariant parameter. At first, the discrete version of our proposed image deblurring and denoising Equation (14) is derived, and the corresponding numerical solution is then given.

Suppose that the noisy image $f\in R^{mn}$ is obtained from a two-dimensional pixel-array with dimension m×n, and $K\in R^{mn\times mn}$ is the discretization of the continuous blurring operator. Now we introduce the discrete version of Equation (14):

(16) $$\min_{u}{∥\nabla u∥}_{1}+\lambda G\left(Ku\right)$$

where $G:R^{mn}\to R$ is defined as

(17) $$G\left(u\right):=\sum_{i}\log\left(1+\frac{\left|u_{i}-f_{i}\right|}{\gamma }\right)+\mu {∥u-g∥}_{2}^{2}.$$

The first term of Equation (16) denotes the discrete total variation of the image *u*, and it is defined as

(18) $${∥\nabla u∥}_{1}:=\sum_{i}\sqrt{{\left(\nabla_{x}u\right)}_{i}^{2}+{\left(\nabla_{y}u\right)}_{i}^{2}}$$

where the discrete gradient $\nabla \in R^{2mn\times mn}$ is given by $\nabla u=\left(\begin{array}{c} \nabla_{x}u\\ \nabla_{y}u \end{array}\right)$.

The first term on the right side of Equation (17) is a robust distance metric, which can be defined as the meridian norm. The meridian norm tends to behave like the $L_{1}$ norm for points within the unitary $L_{1}$ ball and gives the same penalization to large sparse deviations as to small clustered deviations [30].

As in [32], we introduce new variables $v\in R^{2mn}$ and $w\in R^{mn}$, and Equation (16) is then clearly equivalent to the following constrained optimization problem:

(19) $$\min_{u,v,w}{∥v∥}_{1}+\lambda G\left(w\right),s.t.\;v\;=\;{\left(v_{x},v_{y}\right)}^{T}\;=\;\nabla u,w\;=\;Ku.$$

To employ the primal-dual algorithm, we study the following optimization problem:

(20) $$\min_{u,v,w\in X}\max_{p,q\in Y}{∥v∥}_{1}+\lambda G\left(w\right)+\langle v-\nabla u,p\rangle +\langle w-Ku,q\rangle$$

where $p\in R^{2mn}$ and $q\in R^{mn}$ are the dual variables, *X* is a real vector space $R^{mn}$, and $Y=\left\{q\in R^{2mn}:{∥q∥}_{\infty }\leq 1\right\}$, where ${∥q∥}_{\infty }$ is defined as ${∥q∥}_{\infty }=\max_{i\in \left\{1,2,\cdots ,mn\right\}}\sqrt{q_{i}^{2}+q_{i+mn}^{2}}$.

Now we apply the primal-dual algorithm to the optimization problem of Equation (20). The primal-dual algorithm is defined through the following iterations:

(21) $$p^{k+1}=\underset{p}{\arg\max}\langle {\bar{v}}^{k}-\nabla {\bar{u}}^{k},p\rangle -\frac{1}{2\sigma }{∥p-p^{k}∥}_{2}^{2}$$

(22) $$q^{k+1}=\underset{q}{\arg\max}\langle {\bar{w}}^{k}-K{\bar{u}}^{k},q\rangle -\frac{1}{2\sigma }{∥q-q^{k}∥}_{2}^{2}$$

(23) $$u^{k+1}=\underset{u}{\arg\min}-\langle \nabla u,p^{k+1}\rangle -\langle Ku,q^{k+1}\rangle +\frac{1}{2\tau }{∥u-u^{k}∥}_{2}^{2}$$

(24) $$v^{k+1}=\underset{v}{\arg\min}{∥v∥}_{1}+\langle v,p^{k+1}\rangle +\frac{1}{2\tau }{∥v-v^{k}∥}_{2}^{2}$$

(25) $$w^{k+1}=\underset{w}{\arg\min}\lambda G\left(w\right)+\langle w,q^{k+1}\rangle +\frac{1}{2\tau }{∥w-w^{k}∥}_{2}^{2}$$

(26) $${\bar{u}}^{k+1}=2u^{k+1}-u^{k}$$

(27) $${\bar{v}}^{k+1}=2v^{k+1}-v^{k}$$

(28) $${\bar{w}}^{k+1}=2w^{k+1}-w^{k}.$$

In the following, we provide details on how to solve them. Since the objective functions of Equations (21)–(23) are quadratic, the update of *p*, *q*, and *u* can be computed efficiently by

(29) $$p^{k+1}=\sigma \left({\bar{v}}^{k}-\nabla {\bar{u}}^{k}\right)+p^{k}$$

(30) $$q^{k+1}=\sigma \left({\bar{w}}^{k}-K{\bar{u}}^{k}\right)+q^{k}$$

(31) $$u^{k+1}=u^{k}+\tau \left(K^{T}q^{k+1}-\mathrm{div}p^{k+1}\right)$$

where the divergence operator $\mathrm{div}=-\nabla^{T}$. The update in Equation (24) can be obtained by applying the soft thresholding operator as

(32) $$v^{k+1}=\underset{v}{\arg\min}{∥v∥}_{1}+\frac{1}{2\tau }{∥v-t^{k}∥}_{2}^{2}=\frac{t^{k}}{\left|t^{k}\right|}\cdot \max\left\{\left|t^{k}\right|-\tau ,0\right\}$$

where $t^{k}=v^{k}-\tau p^{k+1}$. The optimality condition for (25) is given by

(33) $$\begin{array}{cc} w^{k+1} & =\underset{w}{\arg\min}\lambda \log\left(1+\frac{\left|w-f\right|}{\gamma }\right)\\ & \;\;\;\;+\frac{\mu \lambda }{2}{∥w-g∥}_{2}^{2}+\langle w,q^{k+1}\rangle +\frac{1}{2\tau }{∥w-w^{k}∥}_{2}^{2}\\ & =\underset{w}{\arg\min}\log\left(1+\frac{\left|w-f\right|}{\gamma }\right)\\ & \;\;\;\;+\frac{1+\mu \lambda \tau }{2\lambda \tau }{∥w-\frac{1}{1+\mu \lambda \tau }\left(\mu \lambda \tau g-\tau q^{k+1}+w^{k}\right)∥}_{2}^{2} \end{array};$$

that is

(34) $$w^{k+1}=\gamma .\mathrm{sgn}(a^{k})\cdot \max\left\{\frac{\left|a^{k}\right|-1+\sqrt{{\left(\left|a^{k}\right|+1\right)}^{2}-\frac{4\lambda \tau }{\gamma^{2}\left(1+\mu \lambda \tau \right)}}}{2},0\right\}+f$$

where $a^{k}=\frac{1}{\gamma \left(1\;+\;\mu \lambda \tau \right)}\left(\mu \lambda \tau g-\tau q^{k+1}+w^{k}\right)-\frac{f}{\gamma }$.

We remark that, if *K* is the identity operator, i.e. the degraded image *f* is not blurred but is only corrupted by noise, there is no need to introduce the primal variable *w* and the dual variable *q*, and the algorithm can be simplified accordingly.

The primal-dual algorithm above to solve the optimization problem of Equation (20) can be summarized in the following table.

The termination condition in Algorithm 1 will be discussed in Section 5.

In the rest of this section, we study the existence of the solution to Equation (20) and the convergence of Algorithm 1.

Define $A=\left(\begin{array}{ccc} -\nabla & I & 0\\ -K & 0 & I \end{array}\right)$, $x=\left(\begin{array}{c} u\\ v\\ w \end{array}\right)$, $y=\left(\begin{array}{c} p\\ q \end{array}\right)$, such that Equation (20) is equivalent to

(35) $$\min_{x\in X}\max_{y\in Y}H\left(x\right)+\langle Ax,y\rangle$$

where $H\left(x\right)={∥v∥}_{1}+\lambda G\left(w\right)$.

**Proposition** **1.** The saddle-point set of Equation (35) is nonempty.

**Proof.**  The proof of the above proposition is the same as that for Proposition 2 of [37]. We remark that we can easily verify that the required conditions in [38] are satisfied for the proposed primal-dual formulation:

(H1): *X* and *Y* are nonempty closed convex sets;

(H2): The objective function (denote $\Phi \left(x,y\right)$ ) of (35) is convex-concave on X×Y in the following sense: for each y∈Y, the function $\Phi \left(\cdot ,y\right)$ is convex, for each x∈X, the function $\Phi \left(x,\cdot \right)$ is concave;

(H3): *X* is bounded, or $y_{0}\in Y$ such that $\Phi \left(x,y_{0}\right)\to +\infty$ when $∥x∥\to +\infty$;

(H4): *Y* is bounded, or $x_{0}\in Y$ such that $\Phi \left(x_{0},y\right)\to +\infty$ when $∥y∥\to +\infty$; Thus, there exists a nonempty convex compact set of saddle-points on X×Y of Equation (35). ☐

The following proposition shows the convergence of Algorithm 1.

**Algorithm 1:** Primal-dual algorithm for solving model (20)
**Initialization:** Given σ>0, τ>0, starting points $p^{0}=0$, $q^{0}=0$, $u^{0}={\bar{u}}^{0}=f$, $v^{0}={\bar{v}}^{0}=\nabla u^{0}$ and $w^{0}={\bar{w}}^{0}=Ku^{0}$, and iteration index k=0**Calculate:** $p^{k+1}$, $q^{k+1}$, $u^{k+1}$, $v^{k+1}$, $w^{k+1}$, ${\bar{u}}^{k+1}$, ${\bar{v}}^{k+1}$ and ${\bar{w}}^{k+1}$ from
$$p^{k+1}=\sigma \left({\bar{v}}^{k}-\nabla {\bar{u}}^{k}\right)+p^{k}$$
$$q^{k+1}=\sigma \left({\bar{w}}^{k}-K{\bar{u}}^{k}\right)+q^{k}$$
$$u^{k+1}=u^{k}+\tau \left(K^{T}q^{k+1}-\mathrm{div}p^{k+1}\right)$$
$$t^{k}=v^{k}-\tau p^{k+1}$$
$$v^{k+1}=\frac{t^{k}}{{∥t^{k}∥}_{1}}\cdot \max\left\{{∥t^{k}∥}_{1}-\tau ,0\right\}$$
$$a^{k}=\frac{1}{\gamma \left(1+\mu \lambda \tau \right)}\left(\mu \lambda \tau g-\tau q^{k+1}+w^{k}\right)-\frac{f}{\gamma }$$
$$w^{k+1}=\gamma .\mathrm{sgn}(a^{k})\cdot \max\left\{\frac{\left|a^{k}\right|-1+\sqrt{{\left(\left|a^{k}\right|+1\right)}^{2}-\frac{4\lambda \tau }{\gamma^{2}\left(1+\mu \lambda \tau \right)}}}{2},0\right\}+f$$
$${\bar{u}}^{k+1}=2u^{k+1}-u^{k}$$
$${\bar{v}}^{k+1}=2v^{k+1}-v^{k}$$
${\bar{w}}^{k+1}=2w^{k+1}-w^{k}.$The iteration is terminated if the termination condition is satisfied; otherwise, set k:=k+1 and return to Step (2).

**Proposition** **2.** Let ${∥A∥}_{2}$ be the operator 2-norm of A , and the iteration of $\left(x^{k},y^{k}\right)$ be defined by Algorithm 1. If $\sigma \tau {∥A∥}_{2}^{2}<1$, then $\left(x^{k},y^{k}\right)$ converges to a saddle point$\left(x^{*},y^{*}\right)$ of primal-dual problem in Equation (35).

**Proof.**  The proposition can be seen as a special case of Theorem 1 in [32]. The conclusion (a) of Theorem 1 in [32] establishes that $\left(x^{k},y^{k}\right)$ is a bounded sequence, so that some subsequence $\left(x^{k_{l}},y^{k_{l}}\right)$ converges to some limit $\left(x^{*},y^{*}\right)$. Observe that the conclusion (b) of Theorem 1 in [32] implies that $\lim_{k\to \infty }\left(x^{k}-x^{k-1}\right)=\lim_{k\to \infty }\left(y^{k}-y^{k-1}\right)=0$, and $x^{k_{l}-1}$ and $y^{k_{l}-1}$ in particular converge, respectively, to $x^{*}$ and $y^{*}$. It follows that the limit $\left(x^{*},y^{*}\right)$ is a fixed point of the iterations of Algorithm 1, hence a saddle-point of our problem. ☐

Since ${∥\nabla ∥}_{2}^{2}\leq 8$ (see [4]), ${∥K∥}_{2}\leq 1$ (see [37]), and ${∥A∥}_{2}^{2}\leq {∥\nabla ∥}_{2}^{2}+{∥K∥}_{2}^{2}+1$ (see [18,39]), ${∥A∥}_{2}^{2}\leq 10$. Therefore, in order to ensure the convergence of our algorithm we just need to choose σ and τ such that στ<0.1.

## 5. Experimental Results and Analysis

In this section, numerical results are obtained by applying our proposed models to blurred images corrupted by alpha-stable noise. We also compare our models with other existing and well-known models.

We take six images—Cameraman (256×256), Peppers (256×256), Lena (256×256), Phantom (256×256), Boat (256×256), and Fruits (256×256)—for experiment and comparison. For further comparison, four objective image quality metrics—the peak signal noise ratio (PSNR) in dB, the measure of structural similarity index (SSIM) [40], the multiscale SSIM (MS-SSIM) [41], and the feature similarity index (FSIM) [42]—are used to measure the performance of the proposed models for the test images. Each of the same experiments is repeated 10 times, so the PSNR, SSIM, MS-SSIM and FSIM values are the averaged results of 10 experiments. The PSNR and SSIM are respectively defined as follows:

(36) $$\mathrm{PSNR}=10\mathrm{lg}\left(\frac{255^{2}mn}{{∥\hat{u}-u∥}_{2}^{2}}\right)$$

(37) $$\mathrm{SSIM}=\frac{2\mu_{\hat{u}}\mu_{u}\left(2\sigma_{\hat{u}u}+c_{2}\right)}{\left(\mu_{\hat{u}}^{2}+\mu_{u}^{2}+c_{1}\right)\left(\sigma_{\hat{u}}^{2}+\sigma_{u}^{2}+c_{2}\right)}$$

where $\hat{u}$ is the restored image, *u* is the original image, $\mu_{\hat{u}}$ and $\mu_{u}$ are their respective mean, $\sigma_{\hat{u}}^{2}$ and $\sigma_{u}^{2}$ are their respective variances, $\sigma_{\hat{u}u}$ is the covariance of them, and $c_{1},c_{2}>0$ are constants. PSNR, SSIM, MS-SSIM, and FSIM are all measures of the performance of an image. A higher PSNR indicates that the better restored image will be picked up, and the SSIM, MS-SSIM, and FSIM values are closer to 1. The characteristic of the restored image is more similar to the original image.

In our numerical simulations, we terminate the algorithm when the relative change of the objective function between two consecutive iterations becomes small enough, i.e.,

(38) $$\frac{E\left(u^{k}\right)-E\left(u^{k+1}\right)}{E\left(u^{k}\right)}<\varepsilon$$

where E(·) denotes the objective function of the proposed Equation (14), and ε>0 is a tolerance. For Algorithm 1, we have found that smaller tolerance values (e.g., $\varepsilon =10^{-4}$) do not consistently improve the relative error as the runtimes increase, so we set $\varepsilon =10^{-3}$ in our numerical experiments.

Since γ depends on the noise level, we take the same value of the parameter found in [30], that is, $\gamma =\frac{f_{\left(0.875\right)}\;-\;f_{\left(0.125\right)}}{2}$ (where $f_{\left(c\right)}$ denotes the *c*th quantile of *f*). We chose σ=τ=0.3 and $\mu \gamma^{2}=1$. In addition, the regularization parameter λ balances the trade-off between the TV regularization term and the data fidelity term. We manually tune it in order to obtain the highest PSNR values of the restored image.

**Figure 2:**
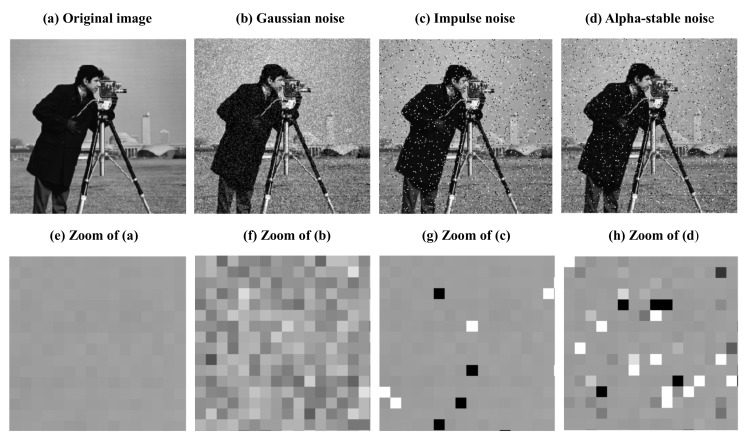
Different noisy images.

We would first like to illustrate the different effects of Gaussian noise, impulse noise, and alpha-stable noise. Figure 2a shows the original Cameraman image, and Figure 2b–d represent, respectively, the images degraded by Gaussion noise, impulse noise, and alpha-stable noise (with α=0.5). Figure 2e–h show the zoomed top left corner of Figure 2a–d.

It is clear from Figure 2 that the image corrupted by Gaussian noise looks different from the images corrupted by impulse noise and alpha-stable noise (with α=0.5), while to some extent the alpha-stable noise and impulse noise are close to each other. For example, some pixels are degraded to white or black with the impulse noise and the alpha-stable noise (with α=0.5), while the image corrupted by Gaussian noise is uniformly modified and all the pixels are corrupted by noise (see Figure 2f). Although the alpha-stable noise is similar to the impulse noise, there are also some very important differences, for instance, in the impulse noise, some pixels are noise-free (see Figure 2g), while in the alpha-stable noise, the noise free pixels are very rare (see Figure 2h). Thus, due to the impulsive character of the alpha-stable noise, we employ the meridian norm in our proposed model.

### 5.1. Image Denoising

In this subsection, we first focus only on the pure denoising case. The noisy image *f* is generated as f=u+η=u+ξρ where ρ follows the alpha-stable distribution, and ξ>0 gives the noise level. We compare the proposed image denoising model with the Cauchy model [18], the TVL1 model [11], and the meridian filter [29]. These models are all efficient for recovering images in impulsive noise.

**Figure 3:**
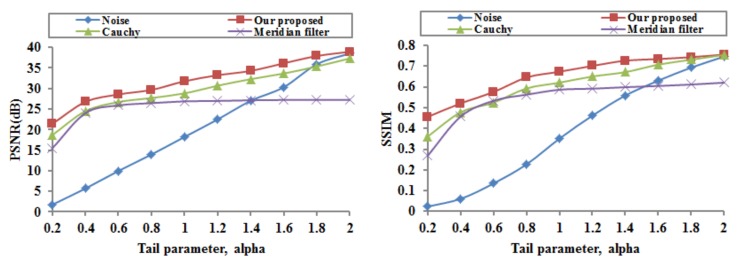
Performances of the noisy image and the recovered images at different alpha parameters.

The proposed image denoising model is applied to the Cameraman image in the presence of alpha-stable noise at different tail parameters α (with ξ=0.04 and ρ following the alpha-stable distribution $S\left(\alpha ,0,0.2,0\right)$). In order to evaluate quantitatively the performances of the proposed image denoising model, two objective criteria, PSNR and SSIM, are computed and provided in Figure 3. The Cauchy and TVL1 models for image denoising perform similarly, so we only provide the results of the Cauchy model in Figure 3.

Figure 3 gives the PSNR and the SSIM of the noisy Cameraman image and the recovered images resulting from the proposed image denoising model, the Cauchy model, and the meridian filter at different tail parameters α. As the tail parameter α increases, the PSNR values and the SSIM values become higher in all of these methods; And as the tail parameter α decreases, the superiority of the proposed method becomes obvious. Moreover, our proposed image denoising model outperforms the Cauchy model and the meridian filter in terms of the PSNR and SSIM at the same tail parameter. In all, the proposed model significantly outperforms the commonly employed image denoising models in impulsive noisy environments (with small α values) while providing comparable performances in less demanding, light-tailed environments (with high α values). In particular, the PSNR values of our proposed model are all above 30 dB at the tail parameter of α≥1, and such values are considered to be perfect recovery results, so we employ the value of ρ, which, in this part, follows the alpha-stable distribution $S\left(1,0,0.2,0\right)$.

For comparison of the performance quantitatively, the PSNR in dB and the SSIM are used to measure the performance of different models for the three noisy test images: Cameraman, Peppers, and Lena. The PSNR values in dB and the SSIM values for noisy images (ξ=0.04 and ρ obeying $S\left(1,0,0.2,0\right)$) and recovered images given by different methods are listed in Table 1.

Table 1 gives the PSNR values and the SSIM values for three different test images and the recovered results of these noisy images resulting from our proposed image denoising model, the Cauchy model, the TVL1 model, and the meridian filter, respectively. Obviously, our proposed image denoising model outperforms the TVL1 model, the Cauchy model, and the meridian filter in terms of the PSNR and SSIM at the same noise levels (ξ=0.04 and ρ following $S\left(1,0,0.2,0\right)$). Take the Cameraman noisy image as an example, with our method, we can increase the PSNR values of the recovered images by 2.836 dB at the same noise levels and obtain the largest SSIM values.

### 5.2. Image Deblurring and Denoising

In the following subsection, we focus on the deblurring and denoising case. Here, we consider the recovery of the blurred images corrupted by both the Gaussian blur (a window size 9×9 and standard deviation of 1) and alpha-stable noise (ξ=0.04). As in the previous subsection, we compare our proposed deblurring and denoising model with other well-known image deblurring and denoising methods for impulsive noise, such as the TVL1 model [11] and the Cauchy model [18].

**Figure 4:**
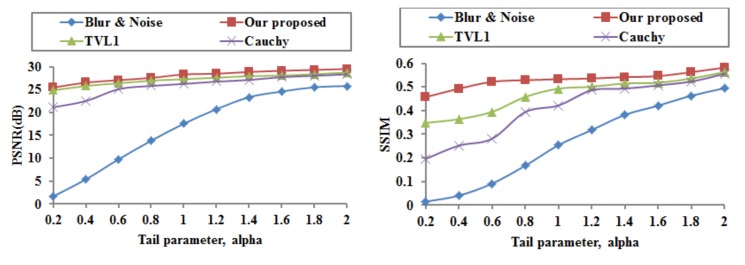
Performances of the blur and noisy images and the recovered images at different tail parameters alpha.

The proposed image deblurring and denoising model is applied to the blurred and noisy Cameraman image at different tail parameters α. The PSNR and SSIM are computed and provided in Figure 4.

Figure 4 provides the quantitative results of our proposed image deblurring and denoising model, the TVL1 model, and the Cauchy model. It is clear that these methods perform well. As the alpha values increase, the PSNR and SSIM values become higher for all these methods. And, as the alpha values decrease, the superiority of our proposed model becomes obvious. Hence, our proposed model has better performance at the same tail parameter α than that of the TVL1 model and the Cauchy model.

Since the PSNR and SSIM performances depend on the tail parameter, it is necessary to choose an appropriate tail parameter for image deblurring and denoising. In the following test, the tail parameter is set to α=1. In practice, we can see from Figure 4 that the recovered results with α=1 are of good quality for all models.

**Figure 5:**
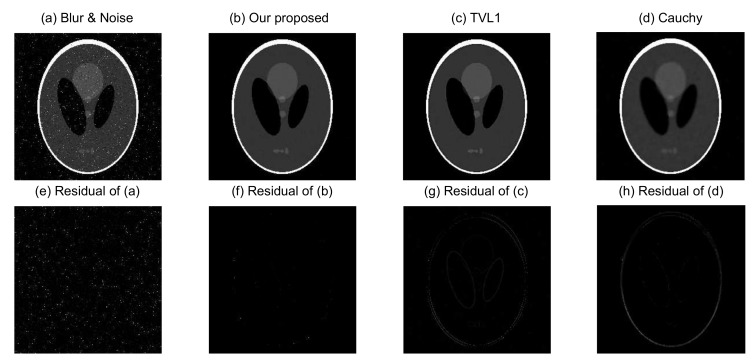
Recovered Phantom images from different methods.

**Figure 6:**
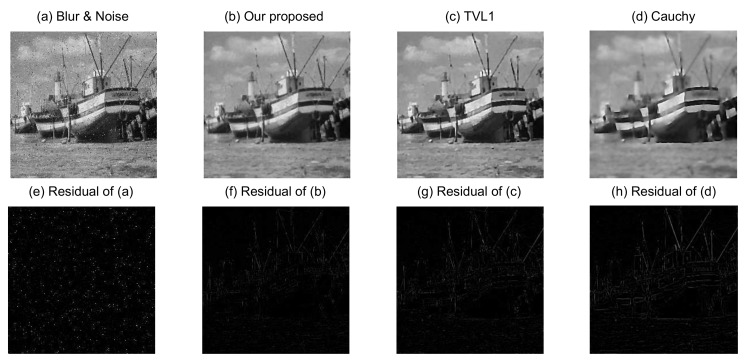
Recovered Boat images from different methods.

**Figure 7:**
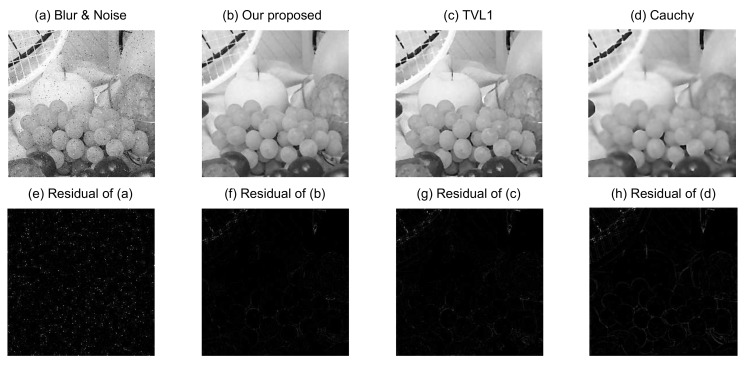
Recovered Fruits images from different methods.

In order to evaluate quantitatively the performance of the proposed image blurring and denoising model, we apply it now to recover three different images (Phantom, Boat, and Fruits) with the Gaussian blur (a window size 9×9 and standard deviation of 1) at the same noise level (ξ=0.04 and ρ following $S\left(1,0,0.2,0\right)$). Experimental results on these test images are shown in Figure 5, Figure 6 and Figure 7, respectively.

Figure 5a is the Phantom blurred and noisy image, and Figure 5b–d are the recovered images from our proposed image blurring and denoising model, the TVL1 model, and the Cauchy model, respectively. The source images in Figure 6 and Figure 7 have similar situations for the Boat and Fruits images, respectively. It is clear from Figure 5, Figure 6 and Figure 7 that the recovered images of our proposed image blurring and denoising model have more detailed information and are much closer to the original test images as compared with the recovered images from the TVL1 model and the Cauchy model.

**Figure 8:**
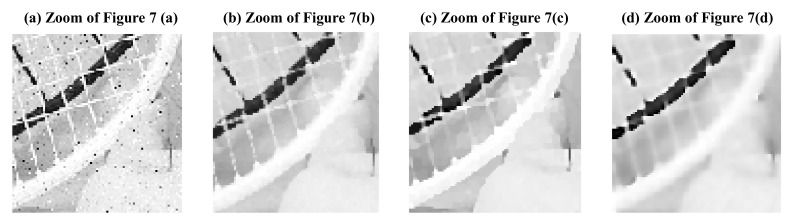
The magnified local regions of the recovered Fruits images from different methods.

Figure 8a–d are the magnified top left regions of Figure 7a–d, respectively. It is clear from Figure 8 that the reconstruction result obtained with our proposed method produces characterizations that are superior to those of the TVL1 and Cauchy methods. We also can see that the restored result of the proposed method can maintain salient features of the line in the original image and has clearer outlines and reduced noise and blur effects.

For further quantitative comparison of the performance of the proposed image deblurring and denoising model, the PSNR in dB and SSIM were computed using the different models for the three different groups of blurred and noisy test images.

The PSNR and SSIM values for blurred and noisy three different test images: Cameraman, Peppers, and Lena (the Gaussian blur with a window size 9×9 and standard deviation of 1, ξ=0.04 and ρ following $S\left(1,0,0.2,0\right)$). The recovered images given by different methods are listed in Table 2.

For easy observation, we took the Fruits image as an example and magnified the top left regions of the restored results with different algorithms. The magnified local regions of the restored results with different algorithms are shown in Figure 8.

In general, larger PSNR values indicate that the recovered image can pick up more information. It is obvious from Table 2 that a notable performance improvement has been achieved by the proposed image deblurring and denoising model as compared with the TVL1 model and the Cauchy model. For example, the PSNRs of the Cameraman image, resulting from the TVL1 model and the Cauchy model are 27.283 dB and 26.244 dB, respectively, while our proposed model gives 28.327 dB, implying that our proposed model provides an improvement of 2.083 dB, as compared with the Cauchy model. This is consistent with the visual effects of Figure 5, Figure 6, Figure 7 and Figure 8.

To further verify the performance of the algorithm, the PSNR, SSIM, MS-SSIM, and FSIM for blurred and noisy Phantom images and recovered images given by different methods are listed in Table 3. It is obvious from Table 3 that a notable performance improvement has been achieved by the proposed image deblurring and denoising model as compared with the TVL1 model and the Cauchy model in terms of these four image quality metrics. This is also consistent with the visual effects of Figure 5. In addition, we have employed other classical test images to evaluate the deblurring and denoising performance and found that a similar performance gain in terms of the PSNR, SSIM, MS-SSIM, and FSIM has been achieved by the proposed method.

## 6. Conclusions

In order to restore images from blur and alpha-stable noise while also preserving their edges, we have proposed a new variational method for restoring blurred images with alpha-stable noise in this paper. Inspired by the ideas of the ROF model and the Cauchy model as in [18], we have obtained a convex model. Theoretical results support the existence and uniqueness of the solution to our proposed model. In addition, we have employed the primal-dual algorithm [32] to solve the corresponding convex problem involved in our proposed model and show that the convergence is guaranteed. Experimental results demonstrate that the proposed method significantly outperforms the commonly employed image deblurring and denoising models in impulsive noisy environments (with small α values, i.e., $\alpha \in \left(0,1.5\right)$), while providing comparable or better performance in less demanding, light-tailed environments (with high α values, i.e., $\alpha \in \left(1.5,2\right)$). 

## Figures and Tables

**Table 1:** The PSNR (dB)/SSIM for noisy images and recovered images.

Models	Different Test Images		
	Cameraman	Peppers	Lena
Noise	18.218/0.351	18.463/0.463	18.372/0.458
Our proposed	31.772/ 0.674	32.869/ 0.856	32.571/ 0.849
TVL1 [11]	28.936/0.623	32.383/0.838	31.959/0.823
Cauchy [18]	28.825/0.621	32.287/0.834	31.853/0.821
Meridian filter [29]	26.883/0.587	30.612/0.812	30.279/0.801

**Table 2:** The PSNR (dB)/SSIM for blurred and noisy images and recovered images.

Models	Different Test Images		
	Cameraman	Peppers	Lena
Blur and Noise	17.556/0.254	18.048/0.391	18.006/0.389
Our proposed	28.327/0.533	29.872/0.766	29.667/ 0.762
TVL1 [11]	27.283/0.501	29.247/0.739	28.971/0.736
Cauchy [18]	26.244/0.472	29.201/0.724	28.583/0.721

**Table 3:** Different image quality metrics for blurred and noisy Phantom images and recovered images.

Models	Different Quality Metrics			
	SSIM	MS-SSIM	FSIM	PSNR
Blur and Noise	0.323	0.741	0.592	18.281
Our proposed	0.981	0.997	0.985	34.623
TVL1 [11]	0.959	0.994	0.921	30.748
Cauchy [18]	0.918	983	0.894	30.292
